# Scutellarein Aggravated Carbon Tetrachloride-Induced Chronic Liver Injury in Gut Microbiota-Dysbiosis Mice

**DOI:** 10.1155/2020/8811021

**Published:** 2020-12-15

**Authors:** Zhimin Miao, Yong Lai, Yingying Zhao, Lingmin Chen, Jianeng Zhou, Chunyan Li, Hai Lan

**Affiliations:** College of Pharmacy, Dali University, Dali 671000, China

## Abstract

Scutellarein (SCU) is an herbal flavonoid, showing hepatoprotective potentials. The study was aimed to investigate whether the hepatoprotective effect of SCU is dependent on the integrity of gut microbiota. Mice received repeated intraperitoneal injections of CCl_4_, followed with or without SCU treatment (15, 30, and 60 mg/kg). Gut microbial community of mice was disrupted by administrating a cocktail of antibiotics (ampicillin, neomycin sulfate, metronidazole, and vancomycin) in drinking water. The results showed SCU plus antibiotics aggravated CCl_4_-induced chronic liver injury, as demonstrated by liver function analysis, histological analysis, and TUNEL assay. SCU activated CYP2E1 expression and worsened CYP2E1-mediated lipid peroxidation and oxidative stress as coadministered with antibiotics. Moreover, when gut microbiota was disrupted by antibiotics, SCU activated I*κ*B*α*/NF-*κ*B pathway and promoted the release of subsequent proinflammatory cytokines including interleukin-6 (IL-6), interleukin-1*β* (IL-1*β*), and tumor necrosis factor-*α* (TNF-*α*). Remarkably, the 16 S rRNA sequencing demonstrated that SCU greatly decreased the relative abundance of *Bifidobacterium* and *Lactobacillus* and increased the relative abundance of *Enterococcus* in gut microbiota-dysbiosis mice. Spearman correlation analysis showed that *Lactobacillus* was positively correlated with SOD and negatively correlated with AST. Collectively, the hepatoprotective effect of SCU is reversed under antibiotics intervention, which may partly involve the activation of CYP2E1 and I*κ*B*α*/NF-*κ*B pathway and diminishment of *Lactobacillus.*

## 1. Introduction

The gut microbiota is a key bridge between host and various environmental factors including diet, heredity, and stress. Recently, mounting evidence suggests that the gut microbiota plays a crucial role in herbal medicine therapies, and it has been proved that a lot of natural products including honokiol and aged citrus peel exert their pharmacological activities via remodeling gut microbiota, such as the enrichment of *Lactobacillus* and *Bifidobacterium,* as well as *Akkermansia*, and the reduction in *Helicobacter* [1, 2]. Therefore, it is not surprising that antibiotics usually deteriorate the beneficial effects of natural products by destroying the gut microbial community.

Chronic liver injury induced by long-term drug therapies, alcohol abuse, and virus infection occurs frequently, arousing great attention in the world. In recent years, bioactive phytochemicals such as flavonoids have attracted considerable attention for hepatoprotection due to their multiple targets and low adverse effects [3]. Scutellarein (SCU) is an herbal flavonoid derived from Chinese traditional herb, Erigeron breviscapus (Vant.) Hand–Mass, and exerts multiple pharmacological effects, such as antioxidation and anti-inflammation, showing hepatoprotective potentials [4, 5]. In healthy volunteers and rats, the oral bioavailability of SCU was found to be merely 2.2% and 10.67%, respectively [4]. The physiological effects of SCU are in notable contrast to its poor bioavailability. So, we hypothesized the gut microbiota might mediate the hepatoprotective effects of SCU.

In the present study, we investigated the effects and mechanisms of SCU on CCl_4_-induced chronic liver injury in gut microbiota-dysbiosis mice.

## 2. Materials and Methods

### 2.1. Drugs and Animals

SCU (purity >98%) was obtained from Yunnan Plant Pharmaceutical Co., Ltd., (Kunming, Yunnan, China). CCl_4_ and antibiotics were purchased from Sinoreagent (Shanghai, China). Male BALB/c mice (6–8 weeks old and 18–22 g) were bred at and obtained from Tianqin Biotechnology Co., Ltd., no.SCXK-2019-0004 (Changsha, Hunan, China). Before experimental treatment, all mice were kept in the specific pathogen-free mouse room for 7 days. All animal experiments were performed in accordance with the guidelines of the Care and Use of Laboratory Animals of the Laboratory Animal Ethical Commission of Dali University (no. 2017–1201) and all efforts were taken to minimize animals suffering.

### 2.2. Experimental Design

Mice were randomly divided into six groups (*n* = 10 per group). (1) Normal group (NA); (2) CCl_4_-treated model group (MA); (3) CCl_4_ and 15 mg/kg SCU cotreatment group (MLA); (4) CCl_4_ and 30 mg/kg SCU cotreatment group (MMA); (5) CCl_4_ and 60 mg/kg SCU cotreatment group (MHA). The drinking water in the NA, MA, MLA, MMA, and MHA groups contains a cocktail of antibiotics (ampicillin (1 g/liter), neomycin sulfate (1 g/liter), metronidazole (1 g/liter), and vancomycin (0.5 g/liter)). (6) CCl_4_ and 60 mg/kg SCU cotreatment group (MH) acts as a positive control and the drinking water in this group was sterile without antibiotics. Chronic liver injury in mice was induced by intraperitoneal injection of 1 ml/kg CCl_4_ (diluted 1 : 9 in olive oil) three times per week. SCU (dissolved in 0.5% CMC-Na) was given by gavage every day. All animals were treated for an additional five weeks. Mice were euthanized and blood samples, stools, and liver tissues were collected 12 h after the final injection of CCl_4_.

### 2.3. Liver Function Measurement

The collected blood samples were centrifuged (3000 rpm, 4°C, 7 min) to obtain serum. The levels of ALT, AST, TBIL, and ALB in the serum were measured according to the manufacturer's instructions (Nanjing Jiancheng Bioengineering Institute, China).

### 2.4. Histological Analysis

Liver samples were fixed in 4% paraformaldehyde for 24 h and then were embedded in paraffin wax. Embedded tissues were cut into 4 *μ*m thick sections and stained with hematoxylin and eosin (H&E) for the histological analyses. Histological score was assessed based on hepatocyte necrosis and hepatic inflammatory cell infiltration.

### 2.5. TUNEL Assay

Hepatocyte apoptosis in the liver tissue was detected by DeadEnd^TM^ Fluorometric TUNEL System according to the manufacture's protocol (Promega, USA). The paraffin-embeded sections were stained with the terminal deoxynucleotidyl transferase dUTP nick end labelling method. The sections were analyzed under a fluorescence microscope and apoptosis area was quantified by Image *J*.

### 2.6. Oxidative Stress Assessment

100 mg snap-frozen liver samples were weighed and homogenized in cold physiological saline. The homogenates were centrifuged (12000 rpm, 4°C, 30 min) to obtain the supernatant. Commercial kits were used to determine the SOD activity and MDA level (Nanjing Iiancheng Bioengineering Institute, China).

### 2.7. Immunohistochemistry (IHC)

Immunohistochemistry for CYP2E1 was performed with the paraffin-embeded liver sections. After blockade of inner peroxidase, sections were incubated with the anti-CYP2E1 antibody (ab28146, 1 : 500, Abcam, Cambridge, UK). Then, sections were incubated with HRP-conjugated secondary antibodies at room temperature for 15 min. Finally, the sections were stained with DAB substrate and counterstained with hematoxylin. Areas of CYP2E1 expression were quantified by Image *J*.

### 2.8. Real-Time Quantitative PCR (RT-qPCR)

Total RNA of 100 mg snap-frozen liver samples was extracted by Trizol reagent (Thermo Fisher Scientific, NY, USA). Total RNA was reverse-transcribed into complementary DNA using RevertAid First Strand cDNA Synthesis Kit according to the manufacture's instructions (Thermo Fisher Scientific, NY, USA). RT-qPCR was operated with a StepOnePlus^TM^ Real-Time PCR system (Thermo Fisher Scientific, NY, USA) in combination with TB Green^®^ Premix Ex Taq^TM^ II (Takara Bio, Inc., Shiga, Japan). The reaction was as follows: a precycling stage at 95°C for 30 s, 40 cycles of denaturation at 95°C for 5 s, and annealing at 60°C for 30 s. The relative expression of mRNA was calculated using the 2^−ΔΔCt^ method and normalized to GAPDH. The PCR primer sequences are listed in Table 1.

### 2.9. Western Blot

Total protein of 100 mg snap-frozen liver tissues was extracted by RIPA lysis buffer and protein concentration was determined by BCA assay kit (Solarbio, Beijing, China) based on the manufacturer's protocols. 40 *μ*g protein was loaded in 10% SDS-PAGE gel and transferred to PVDF membranes. Rabbit anti-I*κ*B*α* (#4812, 1 : 1000, Cell Signaling Technology, Inc., Danvers, MA, USA), anti-NF-*κ*B P65 (#8242, 1 : 1000, Cell Signaling Technology, Inc., Danvers, MA, USA), anti-CYP2E1 (ab28146, 1 : 2000, Abcam, Cambridge, UK), anti-Histone H3 (#4499, 1 : 2000, Cell Signaling Technology, Inc., Danvers, MA, USA), and anti-GAPDH (ab9484, 1 : 2500, Abcam, Cambridge, UK) antibodies were used and goat anti-rabbit IgG H&L (HRP) (ab205718, 1 : 5000, Abcam, Cambridge, UK) as the secondary antibody. The membranes were visualized by ECL reagents, and the protein bands were detected by G:BOX gel imaging system (Syngene, Cambridge, UK).

### 2.10. 16 S rRNA Gene Sequence Analysis

The sequencing serve was provided by Personal Biotechnology Co., Ltd. (Shanghai, China). Fresh feces samples were collected and the total DNA was isolated using QIAamp DNA Stool Kit (Qiagen, Valencia, USA). The purity and concentration of DNA was measured by NanoDrop 2000 ultraviolet spectrophotometer. The bacterial 16 S rRNA gene V3–V4 region was amplified by PCR using the forward primer (338F: 5′-ACTCCTACGGGAGGCAGCA-3′) and the reverse primer (806R: 5′-GGACTACHVGGGTWTCTAAT -3′). The gut microbiota composition was analyzed on an IlluminaMiSeq platform. The alpha diversity including Chao1 and Shannon index was calculated using OUT in QIIME (Denver, USA). The beta diversity was visualized by Principal Coordinate Analysis (PCoA). The genera changes were measured using *Z* score. The correlation between genera and liver injury indicators was analyzed using spearman correlation analysis. All the figures were performed by Personalbio GenesCloud (https://www.genescloud.cn/chart/). The raw data were deposited into NCBI Sequence Read Archive (SRA) database.

### 2.11. Statistical Analysis

All data are expressed as the mean ± SD. Comparisons between groups were assessed by a one-way analysis of variance (AVONA), and Dunnett's test was employed as a post hoc test. *P* < 0.05 was considered significant.

## 3. Results

### 3.1. Effects of SCU plus Antibiotics on Hepatic Injury and Apoptosis

**Figure 1:**
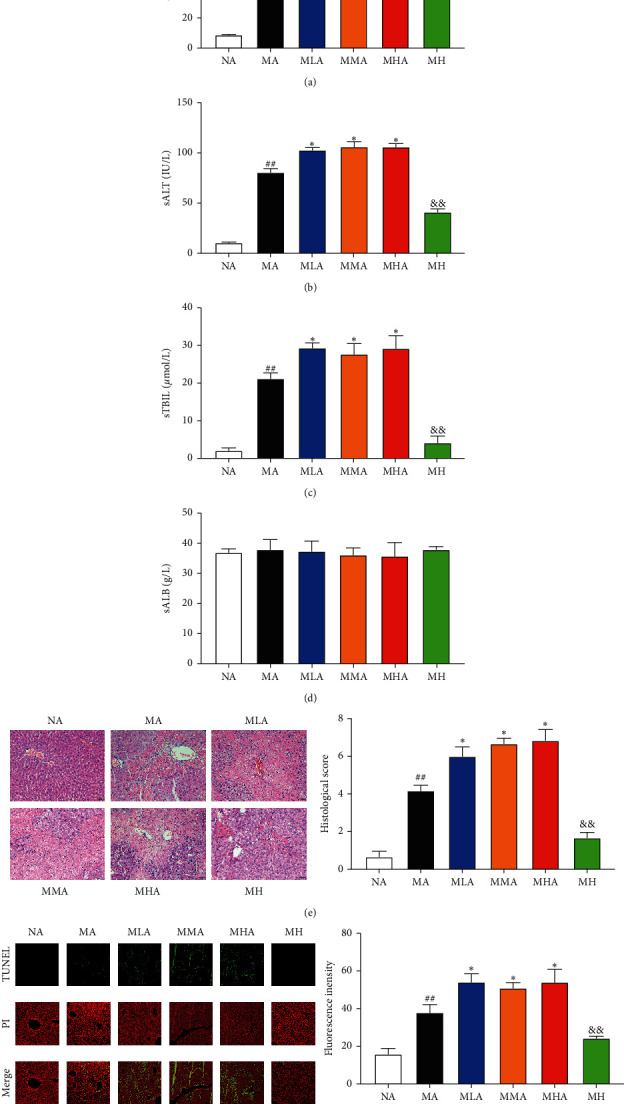
Effects of SCU on hepatic injury and apoptosis. (a) Serum aspartate aminotransferase (AST), (b) alanine aminotransferase (ALT), (c) total bilirubin (TBIL), and (d) albumin (ALB) levels. (e) Liver histology (hematoxylin and eosin (H&E)) of each group, scale bar 50 *μ*m, magnification 200×. (f) TUNEL assay in the livers from different treatment groups, scale bar 100 *μ*m, magnification 200×. The data and error bars are presented as mean ± SD (*n* = 5). ^##^*P* < 0.01 vs. NA group. ^*∗*^*P* < 0.05 vs. MA group. ^&&^*P* < 0.01 vs. MHA group.

The effects of SCU in gut microbiota-dysbiosis mice were first assessed by serum biochemical parameters (ALT, AST, TBIL, and ALB) and 60 mg/kg SCU without antibiotics intervention (MH group) acts as a positive control. As shown in Figures 1(a)–1(c), serum ALT, AST, and TBIL levels were markedly elevated in the MA group with respect to the NA group. Interestingly, serum ALT, AST, and TBIL levels of the SCU plus antibiotics groups were significantly higher than those of the MA group. ALB levels had no significant difference among all groups (Figure 1(d)). Histological observation and TUNEL assay showed that SCU plus antibiotics treatment had higher histological score and hepatocyte apoptosis areas (Figures 1(e) and 1(f)), which was consistent with the results of serum parameters.

### 3.2. Effects of SCU plus Antibiotics on CYP2E1-Mediated Oxidative Stress

**Figure 2:**
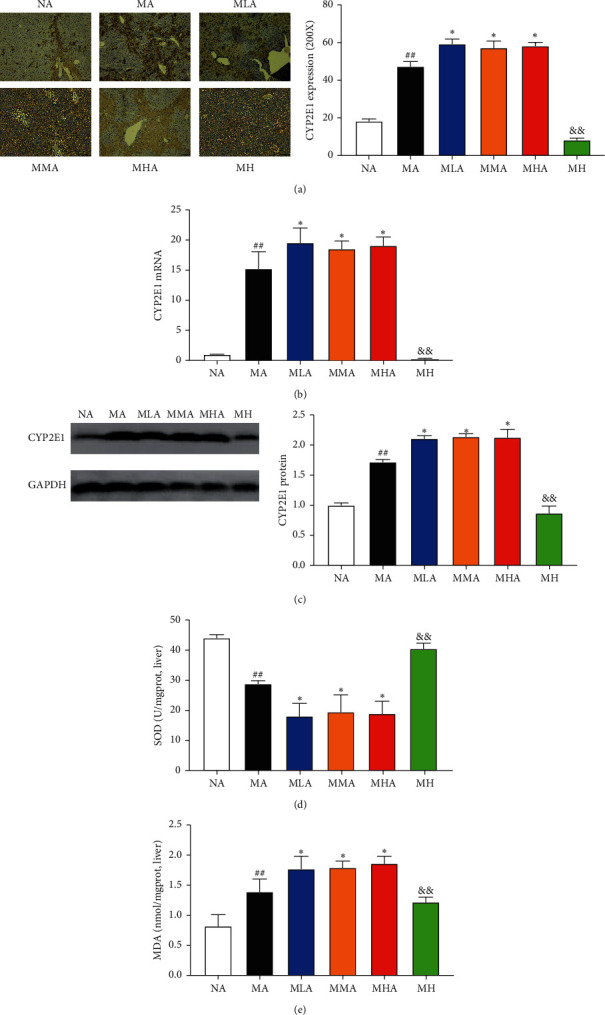
Effects of SCU plus antibiotics on CYP2E1-mediated oxidative stress. (a) Immunohistochemistry analysis of CYP2E1. (b) Relative expression of CYP2E1 mRNA in the liver tissues. (c) Western blotting analysis of CYP2E1. GAPDH was used as the loading control. Quantification of the relative protein levels of CYP2E1. (d) Superoxide dismutase (SOD) activity and (e) malondialdehyde (MDA) level. The data and error bars are presented as mean ± SD (*n* = 5). ^##^*P* < 0.01 vs. NA group. ^*∗*^*P* < 0.05 vs. MA group. ^&&^*P* < 0.01 vs. MHA group.

CCl4 was mainly metabolized by hepatic CYP2E1 to yield free radical, which can irreversibly oxidize biological phenomenon, resulting in lipid peroxidation and oxidative stress, leading ultimately to hepatotoxicity [6]. As shown in Figures 2(a)–2(c), CCl_4_ challenge dramatically increased both the mRNA and protein expression of CYP2E1 in the liver. Treatment of SCU plus antibiotics did not inhibit but activate the mRNA and protein expression of CYP2E1. Then, we measured the hepatic levels of SOD and MDA to quantify oxidative liver injury. As shown in Figures 2(d) and 2(e), compared with the NA group, SOD activity was decreased significantly, and MDA level was increased significantly in the MA group. SCU plus antibiotics treatment showed severe lipid peroxidation and oxidative stress, as proved by higher MDA level and lower SOD activity.

### 3.3. Effects of SCU plus Antibiotics on I*κ*B*α*/NF-*κ*B Pathway-Mediated Inflammation Response

**Figure 3:**
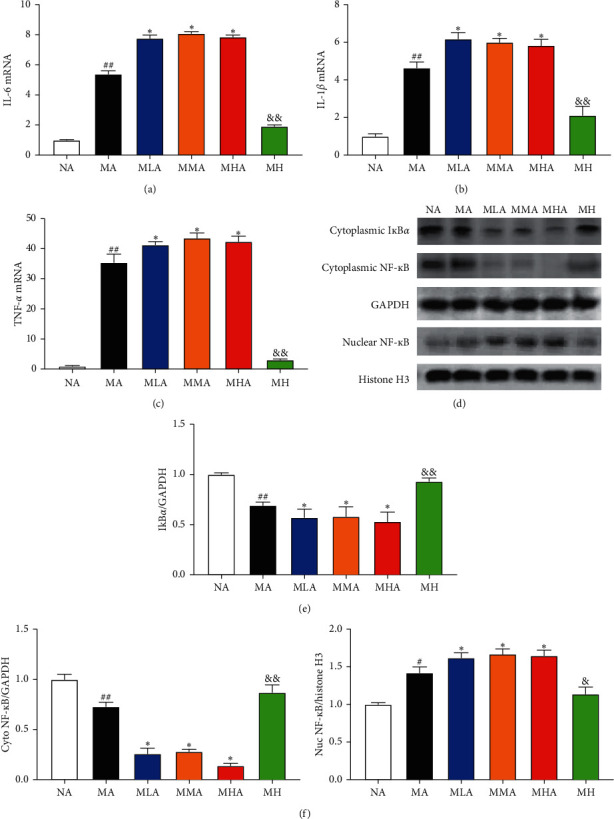
Effects of SCU plus antibiotics on I*κ*B*α*/NF-*κ*B pathway-mediated inflammation response. Relative expression of (a) IL-6, (b) IL-1*β,* and (c) TNF-*α* mRNA in the liver tissues. (d) Western blotting analysis of I*κ*B*α* and NF-*κ*B. GAPDH and Histone H3 were used as the loading control. Quantification of the relative protein levels of (e) I*κ*B*α* and (f) NF-*κ*B. The data and error bars are presented as mean ± SD (*n* = 5). ^#^*P* < 0.05; ^##^*P* < 0.01 vs. NA group. ^*∗*^*P* < 0.05; ^*∗∗*^*P* < 0.01 vs. MA group. ^&^*P* < 0.05; ^&&^*P* < 0.01 vs. MHA group.

Oxidative stress is suggested a trigger of cell inflammation, we then assessed the IL-6, IL-1*β,* and TNF-*α* mRNA levels that are the key inflammatory cytokines in the liver tissues by RT-qPCR. As shown in Figure 3(a), CCl_4_ challenge dramatically increased the IL-6, IL-1*β,* and TNF-*α* mRNA levels in the liver tissue. However, the IL-6, IL-1*β,* and TNF-*α* mRNA levels were even higher in the SCU plus antibiotics groups. Activation of I*κ*B*α*/NF-*κ*B pathway has been shown to induce the production of cytokines, thus we measured the I*κ*B*α* and NF-*κ*B expression levels in the liver. As shown in Figures 3(d)–3(f), the expression of I*κ*B*α* in the MA group was decreased relative to the NA group, but this trend was exacerbated by SCU plus antibiotics cotreatment. In the MA group, we detected reduced cytoplasmic NF-*κ*B and increased nuclear NF-*κ*B expression relative to the NA group. However, SCU plus antibiotics cotreatment aggravated these changes in NF-*κ*B levels caused by repeated injections of CCl4.

### 3.4. Effects of SCU plus Antibiotics on Gut Microbiota

**Figure 4:**
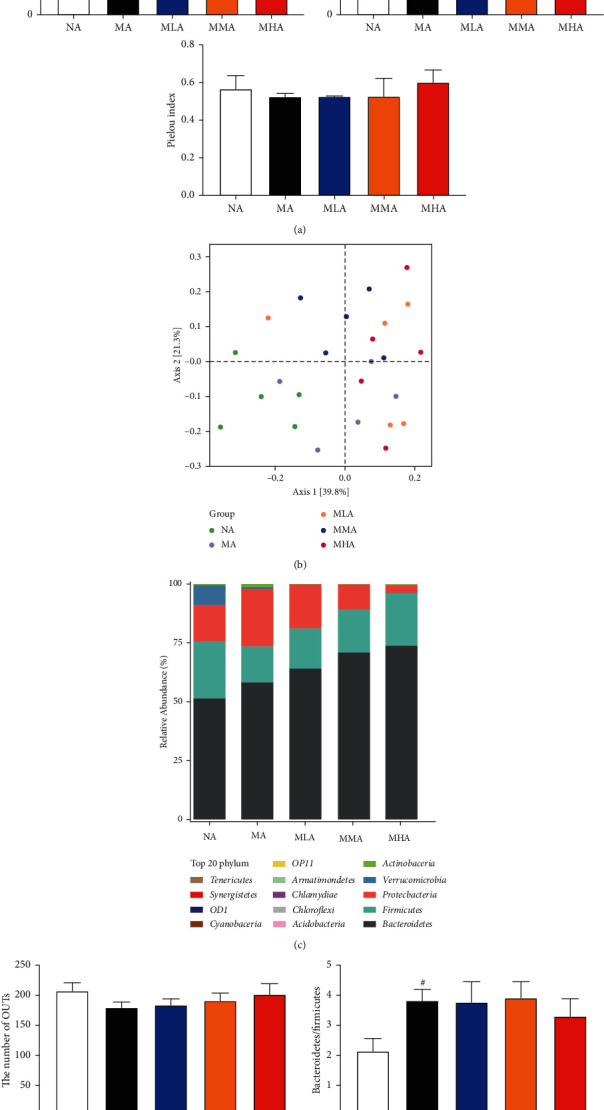
Effects of SCU plus antibiotics on gut microbiota. (a) *α*-Diversity of the gut microbiota. (b) *β*-Diversity of the gut microbiota. (c) Relative abundance of different phyla in the gut microbiota of mice. (d) The top two gut microbial communities at phylum level. The data and error bars are presented as mean ± SD (*n* = 5). ^#^*P* < 0.05 vs. NA group.

We sought to determine the effect of SCU plus antibiotics on gut microbiota diversity and compositions. We analyzed the gut microbiota in feces samples from all groups by 16 S rRNA sequencing. Microbiota community diversity was first evaluated by *α*-diversity employing indices including Chao1, Shannon, and Pielou indices that represent the richness, diversity, and uniformity of gut microbiota, respectively. *β*-Diversity was used to compare the similarity of overall community structure, which employed an unsupervised multivariate statistical assessment such as PCoA. As shown in Figure 4(a), Chao1, Shannon, and Pielou indices had no significant difference among five groups. As shown in Figure 4(b), the structure of gut microbiota in the MA group was more similar to that of the NA group. Furthermore, taxon-based analysis at phylum level was preliminarily used to assess the composition of gut microbiota from all groups (Figure 4(c)). As shown in Figure 4(d), the *Bacteroidetes*/*Firmicutes* radio in the MA group was significantly increased relative to the NA group. However, the *Bacteroidetes*/*Firmicutes* radio had no significant difference between the MA group and SCU plus antibiotics groups.

### 3.5. Effects of SCU plus Antibiotics on Key Phylotypes of Gut Microbiota

**Figure 5:**
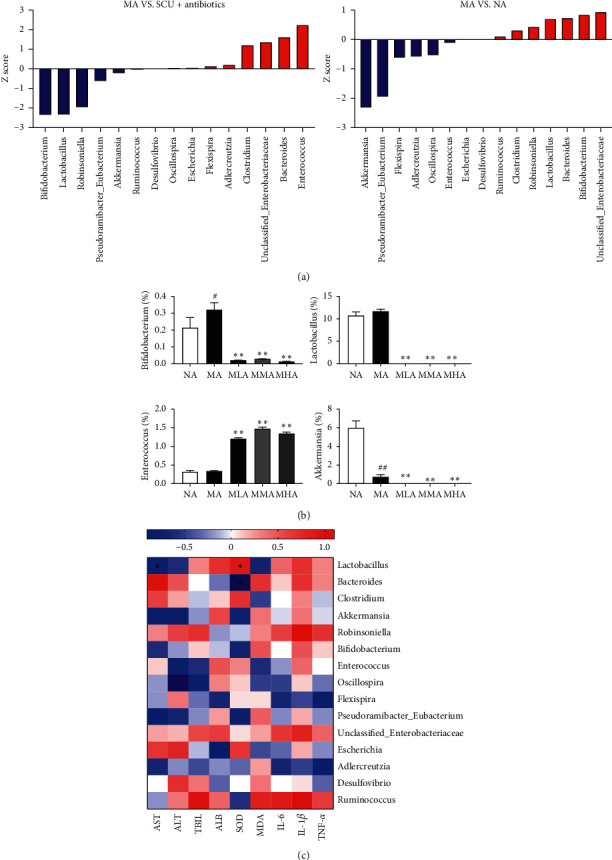
Effect of SCU plus antibiotics on key phylotypes of gut microbiota. (a) The genera changes were assessed by Z score. (b) Significant difference was found in four genera. (c) Heatmap of the correlations between gut microbiota and liver injury indicators. ^∗^indicates a significant correlation (*P* < 0.05). The data and error bars are presented as mean ± SD (*n* = 5). ^#^*P* < 0.05; ^##^*P* < 0.01 vs. NA group. ^*∗∗*^*P* < 0.01 vs. MA group.

We sought to determine which bacterial members of the microbiome were responsible for the observed reversed hepatoprotective effect of SCU. We performed *Z* score to assess the changes of 15 genera that showed high abundance (Figure 5(a)). The relative abundance of *Bifidobacterium* and *Lactobacillus* was significantly increased and the relative abundance of *Enterococcus* was significantly decreased in the MA group relative to the SCU plus antibiotics groups. The relative abundance of *Akkermansia was* significantly decreased in the MA group than that in the NA group (Z score >2) (Figure 5(b)). Moreover, we investigated the relationship between the 15 genera and liver injury indicators (AST, ALT, TBIL, ALB, SOD, MDA, IL-6, IL-1*β,* and TNF-*α*) by using spearman correlation analysis, and the result demonstrated that *Lactobacillus* was positively correlated with SOD and negatively correlated with AST. *Bacteroides* was negatively correlated with SOD (Figure 5(c)).

## 4. Discussion

The gut microbiota plays a crucial role in mediating the pharmacological activities of various natural products. Many flavonoids have been reported to exhibit their beneficial effects by modulating the gut microbiota [7–10]. In this study, we used a cocktail of antibiotics to disrupt the gut microbiota and observed the alteration of hepatoprotective effects of SCU on CCl_4_-induced chronic liver injury.

Serum ALT and AST levels are the preferred indicators for evaluation of liver function. CCl_4_-induced hepatocellular damage and the subsequent rupture of the plasma membrane cause the release of intracellular enzymes such as ALT and AST into the systemic circulation, thereby increasing serum ALT and AST levels [11]. In our study, CCl_4_ intoxication induced a profound elevation in the serum ALT and AST levels. However, these transaminases were elevated when SCU is cotreated with antibiotics. Elevated serum bilirubin levels may be caused by release of unconjugated or conjugated bilirubin from injured hepatocytes [12]. Serum TBIL level was dramatically increased by CCl_4,_ which was still maintained high level in the SCU plus antibiotics groups. The liver is the exclusive site of synthesis of ALB and serum ALB serves as a true test of hepatic synthetic function [12]. However, serum ALB has a very long half-life, which may not be affected in the short-term experimental liver injury model. Our results demonstrated that the serum ALB level had no significant difference among all groups. Histological analysis and TUNEL assay can reflect pathological process and hepatocyte apoptosis level, respectively. The results demonstrated that hepatocyte necrosis, inflammatory cell infiltration, and hepatocyte apoptosis were severe in the SCU plus antibiotics treatment groups relative to the MA group. Taken together, the hepatoprotective effect of SCU was reversed in gut microbiota-dysbiosis mice.

Hepatic CYP2E1 is mainly responsible for the metabolism of CCl_4_ to produce highly-reactive trichloromethyl free radicals and plays a vital role in the regulation of CCl_4_-induced lipid peroxidation, oxidative stress, and inflammatory response [13, 14]. CCl_4_-derived free radical can attack hepatocyte membranes, leading to hepatocyte necrosis [15]. The results showed that 60 mg/kg SCU exerted potent inhibitory effect on CYP2E1, which was reversed by antibiotics intervention. CCl_4_-derived free radical can irreversibly oxidize biological macromolecules including DNA, proteins, and lipids, leading ultimately to hepatotoxicity [15]. MDA is the final product of lipid peroxidation and SOD is one of the most important antioxidant enzymes with high endogenous expression in the liver that eliminates toxic free radicals and reactive toxic CCl_4_ metabolites [16]. The MDA level and SOD activity can reflect the levels of oxidative stress. In our study, SCU worsened lipid peroxidation and oxidative stress, as evidenced by higher MDA levels and lower SOD activity. Oxidative stress is suggested a trigger of inflammation. In this study, we evaluated the key inflammatory cytokines (IL-6, IL-1*β,* and TNF-*α* mRNA) in the liver tissues and the resulted showed that 60 mg/kg could significantly reduce the mRNA levels of them, showing potent anti-inflammatory capacity. However, the anti-inflammatory activity of SCU was reversed under antibiotics intervention, as proved by higher mRNA levels of IL-6, IL-1*β,* and TNF-*α*. CCl_4_-induced oxidative stress triggers the degradation of I*κ*B*α* to increase the DNA-binding affinity of NF-*κ*B. Then, NF-*κ*B can be activated for translocation from the cytoplasm to the nucleus to regulate the expression of multiple target genes including IL-6, IL-1*β,* and TNF-*α* to exacerbate hepatic damage [17, 18]. The results showed that 60 mg/kg SCU significantly restored I*κ*B*α* degradation and suppressed NF-*κ*B activation which was reversed by SCU plus antibiotics. These results suggested that the antioxidant and anti-inflammatory activities of SCU were reversed in gut microbiota-dysbiosis mice.

The gut microbiota can be deemed as an important part of host body that provides additional benefits such as extraction of calories from otherwise indigestible carbohydrates, metabolism of bile acid, and exogenous drugs, among others [19]. Intestinal microflora dysbiosis is an established concept contributing to liver disease. Disruption of gut microbiota can lead to a variety of liver diseases such as nonalcoholic steatohepatitis [20]. Dysbiosis following the toxic liver injury is characterized by an increased prevalence of the phylum Firmicutes, namely, member in the genus *Lactobacillus*. Moreover, there is a significant increase in the number of OUTs in the phylum Actinobacteria in the toxin-treated group [21]. In this study, we demonstrated that experimental dysbiosis induced by antibiotics resulted in a slight increase in liver injury. In the MA group, we found the relative abundance of *Lactobacillus* and Actinobacteria were increased, which was consistent with the previous work [21]. Although the malfunction of gut microbiota occurred under antibiotics intervention, *Lactobacillus* still showed apparent correlation with AST and SOD. *Lactobacillus* has been widely reported to possess beneficial effects to the liver. Shi et al [22] and Chen et al [23] have proved that supplementation of *Lactobacillus* can effectively attenuate CCl_4_-induced liver injury. Saeedi et al. [24] have demonstrated that *Lactobacillus*-derived 5-methoxyindoleacetic acid could activate hepatic Nrf2 to protect against oxidative liver injury. However, the relative abundance of *Lactobacillus* was significantly decreased in the SCU plus antibiotics groups, which might partly account for reversed hepatic effects of SCU. Dysbiosis might result in intestinal inflammation, disruption of the gut barrier, and bacterial translocation [25, 26]. The translocated bacterial products (endotoxin) induce hepatic inflammation and liver injury [27]. However, bacterial products translocation depends on the degree of gut leakiness. Fouts et al. [28] have proved that gut leakiness is lower following toxin-induced liver injury relative to other types of liver damage such as cholestasis. Thus, intestinal microflora dysbiosis might not contribute as much to the progression of toxic liver injury. The bioavailability of SCU is supposed to be very low and SCU is very likely to be degraded by gut microbes to generate metabolites with more pharmacological activity and higher bioavailability. Shi [29] et al. have reported that the main metabolites of SCU are scutellarein and 6-O-methylscutellarein *in vivo*. Furthermore, 6-O-methylscutellarein showed potent antithrombotic activity, stronger antioxidant activity, and balanced solubility and permeability compared with SCU, which warrants further investigation.

## 5. Conclusion

Collectively, our studies demonstrated that the hepatoprotective effect of SCU is reversed under antibiotics intervention, which may partly involve the activation of CYP2E1 and I*κ*B*α*/NF-*κ*B pathway and diminishment of *Lactobacillus*.

## Figures and Tables

**Table 1:** Primers for real-time PCR.

Genes	Forward primers (5′-3′)	Reverse primers (5′-3′)	Accession number
IL-6	CTGCAAGAGACTTCCATCCAG	AGTGGTATAGACAGGTCTGTTGG	NM_031168
IL-1*β*	TGTGAAATGCCACCTTTTGA	GGTCAAAGGTTTGGAAGCAG	NM_008361
TNF-*α*	CAGGCGGTGCCTATGTCTC	CGATCACCCCGAAGTTCAGTAG	NM_013693
CYP2E1	TTTCCCTAAGTATCCTCCGTGAC	CTTAATCGAAGCGTTTGTTGA	NM_021282
GAPDH	GGTTGTCTCCTGCGACTTCA	TGGTCCAGGGTTTCTTACTCC	NM_008084

## Data Availability

The data used to support the findings of the current study are included in this published article.

## Abbreviations

SCU: Scutellarein
CCl_4_: Carbon tetrachloride
CMC-Na: Sodium carboxymethyl cellulose
ALT: Aminotransferase
AST: Aspartate aminotransferase
TBIL: Total bilirubin
ALB: Albumin
MDA: Malondialdehyde
SOD: Superoxide dismutase
IL-1*β*: Interleukin-1*β*
IL-6: Interleukin-6
TNF-*α*: Tumor necrosis factor-*α*
H&E: Hematoxylin and eosin
IHC: Immunohistochemistry.
